# Cellular and Molecular Mechanisms in the Pathogenesis of Multiple Sclerosis

**DOI:** 10.3390/cells9102223

**Published:** 2020-10-01

**Authors:** Edwin C. K. Wan

**Affiliations:** 1 Department of Microbiology, Immunology, and Cell Biology, West Virginia University, Morgantown, WV 26506, USA; edwin.wan@hsc.wvu.edu; 2 Department of Neuroscience, West Virginia University, Morgantown, WV 26506, USA; 3 Rockefeller Neuroscience Institute, West Virginia University, Morgantown, WV 26506, USA

Multiple sclerosis (MS) is one of the most common neurological disorders in young adults. The etiology of MS is not known, but it is generally accepted that it is autoimmune in nature. Our knowledge of the pathogenesis of MS has increased tremendously in the past decade through clinical studies and the use of experimental autoimmune encephalomyelitis (EAE), a model that has been widely used for MS research. Major advances in the field, such as understanding the roles of pathogenic Th17 cells, myeloid cells, and B cells in MS/EAE, as well as cytokine and chemokine signaling that controls neuroinflammation, have led to the development of potential and clinically approved disease-modifying agents (DMAs).

There are many aspects related to the initiation, relapse and remission, and progression of MS that are yet to be elucidated. For instance, what are the genetic and environmental risk factors that promote the initiation of MS and how do these factors impact the immune system? What factors drive the progression of MS and what are the roles of peripheral immune cells in disease progression? How do the CNS-infiltrated immune cells interact with the CNS-resident glial cells when the disease progresses? What is the role of the microbiome in MS? Can we develop animal models that better represent subcategories of MS? Understanding the cellular and molecular mechanisms that govern the pathogenesis of MS will help to develop novel and more specific therapeutic strategies that will ultimately improve clinical outcomes of the treatments. This Special Issue of *Cells* has published original research articles, retrospective clinical reports, and review articles that investigate the cellular and molecular basis of MS.

Most DMAs can effectively reduce the rate of relapse in patients with relapsing–remitting MS (RRMS) but these DMAs are ineffective in stopping the disease from progressing. Ozanimod is a sphingosine-1-phosphate (S1P1 and S1P5) receptor agonist that is recently approved for the treatment of clinically isolated syndrome, RRMS, and active secondary progressive MS (SPMS). Since neurodegeneration is a prominent feature for SPMS, Centonze and colleagues [1] sought to investigate the potential neuroprotective effect of ozanimod. Using corticostriatal slices prepared from EAE-induced mice, they showed a direct anti-excitotoxic effect of ozanimod and found that ozanimod reduces the expression of inflammatory marker iNOS, IL-1β, and TNF. The authors further demonstrated that ozanimod reduces the synaptotoxic effect during EAE by modulating lymphocytes. To further investigate whether the effect of ozanimod is due to the modulation of S1P1 or S1P5 activity, the authors used selective S1P1 and S1P5 agonists and showed that targeting S1P1 is more effective than targeting S1P5 in ameliorating glutamatergic dysfunction. Finally, they showed that mice treated with S1P1 agonist developed less severe EAE. Overall, this study provides evidence that targeting S1P1/S1P5 activity has both anti-inflammatory and neuroprotective effects. 

Inflammation in the CNS during relapse can be caused by mediators produced from both peripheral infiltrated immune cells and CNS-resident glial cells. While most current DMAs for MS treatment target blocking the infiltration and/or function of peripheral immune cells, the potential of targeting glial cells in MS is not well explored. Michetti, Ria, and colleagues [2] showed that targeting S100B, a protein primarily produced by astrocytes, can ameliorate the relapsing–remitting EAE. EAE-induced mice treated with pentamidine isethionate (PTM), an antiprotozoal drug shown previously to block S100B activity, had reduced EAE severity score in the preclinical, onset, and remission phases during the disease course, which correlates with the reduction of *Ifng* and *Tnfa* expression in the brain, as well as the decrease of NOS activity. The numbers of CD68+ cells and demyelinating lesions were reduced in the PTM-treated EAE mice compared to EAE mice without drug treatment. Overall, this study suggests that targeting neurotoxic mediators produced by astrocytes reduces the severity of EAE. Whether the same treatment strategy applies to MS warrants further investigation. 

Demyelination is a hallmark of MS pathology. NG2-glia are oligodendrocyte precursors that can differentiate into mature oligodendrocytes and thus may contribute to remyelination in patients with MS. Using a sophisticated StarTrack approach to follow the fate of NG2-glia which are derived from single progenitors from the dorsal subventricular zone, Lopez-Mascaraque and colleagues [3] investigated the phenotypic heterogenicity of NG2-glia relative to their ontogenic origin, and asked whether EAE triggers a clonal NG2-glial response. They showed that the NG2-glia from single progenitors dispersed throughout the grey and white matter in a clonal manner. In addition, the heterogeneity of NG2-glia seems established in the early embryonic development stage. Using EAE as the disease model, the authors further showed that the clonal NG2-glia are morphologically diverse relative to the distance from EAE lesions. Overall, this study improves our understanding in the nature of heterogeneity of NG2-glia under normal and pathological conditions. 

Although the activation of autoreactive T cells plays a critical role in the pathogenesis of MS, the etiology of this disease remains uncertain. The “inside-out” theory proposes that MS is initiated by demyelination of the CNS, followed by the activation of adaptive immune response in the periphery. Coorssen, Mahns, and colleagues [4] investigated whether the cuprizone-mediated demyelination in mice, in the presence of pertussis toxin, stimulates T-cell infiltration to the brain. They showed that both standard-dose (0.2%) and low-dose (0.1%) treatment of cuprizone for five weeks induces oligodendrocytosis, demyelination, and gliosis in the brain. However, standard-dose treatment also induces splenic atrophy and a more significant reduction of CD4+ and CD8+ cells in the spleen compared to mice with low-dose cuprizone treatment. They further showed that demyelination in the brain does not stimulate T-cell infiltration, even in the presence of pertussis toxin, which is known to disrupt blood–brain barrier integrity. In addition, the authors determined changes in the brain proteome following cuprizone treatment using two-dimensional gel electrophoresis coupled with liquid chromatography and tandem mass spectrometry. They found changes of multiple brain proteoforms following cuprizone treatment, the majority of which are associated with mitochondrial function. Thus, their study suggests that the cuprizone-induced demyelination may be elicited by mitochondrial perturbations. 

In a clinical prospective, identification of biomarkers in patients’ blood may serve as a simple and rapid tool to evaluate disease activity in MS patients. D’Amico and colleagues [5] performed a retrospective study to analyze data from patients with RRMS, and asked whether the blood neutrophil-to-lymphocyte ratio (NLR) correlates with disease severity in these patients. The authors obtained NLR data from 84 newly diagnosed, naïve RRMS patients and found that NLR ratio positively correlates with high disease activity, classified as at least two relapses in the year prior to the study entry and at least one gadolinium-enhancing lesion at the time of study. This study, although with a small sample size, suggests that NLR may serve as one of the biomarkers to evaluate disease activity in MS patients. This observation should be confirmed further with multicenter, large cohort clinical studies. 

In addition to the aforementioned research articles, this Special Issue of *Cells* also published three review articles that cover different research topics in MS. Forsthuber and colleagues [6] summarized the role of memory CD4+ T cells in autoimmune diseases, with specific focus on MS and EAE. Vecsei and colleagues [7] discussed the importance of kynurenines in the pathogenesis of MS. Last but not least, we [8] provided a comprehensive review on the role of granulocyte-macrophage colony-stimulating factor (GM-CSF) in mediating neuroinflammation, and discussed the potential of targeting GM-CSF in the treatment of MS. 

Taken together, articles published in this Special Issue of *Cells* have covered a variety of topics in MS, which help us to better understand the underlying cellular and molecular mechanisms that drive MS pathogenesis. 
